# Epidemiology, Pathogenesis, and Control of a Tick-Borne Disease- Kyasanur Forest Disease: Current Status and Future Directions

**DOI:** 10.3389/fcimb.2018.00149

**Published:** 2018-05-09

**Authors:** Syed Z. Shah, Basit Jabbar, Nadeem Ahmed, Anum Rehman, Hira Nasir, Sarooj Nadeem, Iqra Jabbar, Zia ur Rahman, Shafiq Azam

**Affiliations:** ^1^Center of Excellence in Molecular Biology, University of the Punjab, Lahore, Pakistan; ^2^Institute of Biochemistry and Biotechnology, University of the Punjab, Lahore, Pakistan; ^3^Center of Biotechnology and Microbiology, University of Peshawar, Peshawar, Pakistan; ^4^School of Biological Sciences, University of the Punjab, Lahore, Pakistan

**Keywords:** KFDV, pathogenesis, transmission, molecular epidemiology, prevention and control, diagnosis, animal models

## Abstract

In South Asia, *Haemaphysalis spinigera* tick transmits Kyasanur Forest Disease Virus (KFDV), a flavivirus that causes severe hemorrhagic fever with neurological manifestations such as mental disturbances, severe headache, tremors, and vision deficits in infected human beings with a fatality rate of 3–10%. The disease was first reported in March 1957 from Kyasanur forest of Karnataka (India) from sick and dying monkeys. Since then, between 400 and 500 humans cases per year have been recorded; monkeys and small mammals are common hosts of this virus. KFDV can cause epizootics with high fatality in primates and is a level-4 virus according to the international biosafety rules. The density of tick vectors in a given year correlates with the incidence of human disease. The virus is a positive strand RNA virus and its genome was discovered to code for one polyprotein that is cleaved post-translationally into 3 structural proteins (Capsid protein, Envelope Glycoprotein M and Envelope Glycoprotein E) and 7 non-structural proteins (NS1, NS2A, NS2B, NS3, NS4A, NS4B, and NS5). KFDV has a high degree of sequence homology with most members of the TBEV serocomplex. Alkhurma virus is a KFDV variant sharing a sequence similarity of 97%. KFDV is classified as a NIAID Category C priority pathogen due to its extreme pathogenicity and lack of US FDA approved vaccines and therapeutics; also, the infectious dose is currently unknown for KFD. In India, formalin-inactivated KFDV vaccine produced in chick embryo fibroblast is being used. Nevertheless, further efforts are required to enhance its long-term efficacy. KFDV remains an understudied virus and there remains a lack of insight into its pathogenesis; moreover, specific treatment to the disease is not available to date. Environmental and climatic factors involved in disseminating Kyasanur Forest Disease are required to be fully explored. There should be a mapping of endemic areas and cross-border veterinary surveillance needs to be developed in high-risk regions. The involvement of both animal and health sector is pivotal for circumscribing the spread of this disease to new areas.

## Introduction

Ticks are known as the most common obligate blood-sucking ectoparasites. In contemporary era, a growing number of cases have been registered and, simultaneously, ticks are spreading worldwide and expanding to new geographical locations. Transmission of many bacterial, viral, and rickettsial diseases happens through ticks as they act as a vector for pathogenic organisms. Their bite is painless, consequently, in significantly a number of cases, they remain unnoticed (Otranto et al., 2014). Till date, more than 800 known species of ticks has been identified yet which are classified into three known families: Argasidae, Ixididae, and Nuttaliellidae (Guglielmone et al., 2010).

Tick borne diseases (TBDs) have been growing and spreading worldwide consequent to the increase in tick-infested areas like forests, grasslands, pastureland, and fields. In addition, different human behaviors including sitting on logs, visiting tick-infested parks and sitting along trees are further adding to the potential risk factors for acquiring tick borne infections (Lane et al., 2004).

Ticks and tick borne pathogens are significantly regulated by climatic and environmental conditions. Biotic and abiotic factors, climatic niche characterization, and tick-pathogen interactions all play an important role in tick distribution across different geographical regions of the world (Filippova et al., 1976). Current and comprehensive listing of tick associated pathogens, their hosts, and geographic distributions across the globe are presented in Table 1.

**Table 1:** Overview of tick-borne diseases caused by possible causative agents i.e., bacteria (B), viruses (V), and protozoa (P) with reference to organism, vector, host, geographical distribution, symptoms, zoonosis (Z), fatality rate, availability of therapeutics, and vaccination.

**S. No**	**Disease**	**B/V/P**	**Organism**	**Vector**	**Host**	**Region**	**Symptoms**	**Z (+/−)**	**F.R. (%)**	**Treatment**	**Vaccine**	**References**
1	Relapsing fever	B	*Borrelia spp*.	*Ornithodoros spp*.	Humans, rodents	Spain, Saudi Arabia, Africa, Asia, Canada, Western united States	High fever, flu, headache, muscle pain, rigors, cough, joint pain, rash	+	1% with treatment, 30–70% without treatment	Penicillin, tetracycline, doxycycline, erythromycin	Not available	Otranto et al., 2014
2	Tularemia	B	*Francisella tularensis*	*D. variabilis, D. andersoni, D. marginatus, I. dentatus, I. ricinus, A. americanum*	Humans, rodents, hares and rabbits	US-Southeast, West, South-central, widespread	Fever, chills, headache, exhaustion, skin ulcer, swollen lymph glands	+	<2% with treatment, 30–60% without treatment	Streptomycin, gentamycin, doxycycline	Live attenuated vaccine is available	Guglielmone et al., 2010
3	Lyme disease or borreliosis	B	*B. mayonii, B. burgdorferi, B. afzelii, B. garinii*	*Ixodes scapularis, I. persulcatus, I. pacificus, I. ricinus*	Humans, pets, livestock	Eurasia, North America	Fever, arthritis, neuroborreliosis, erythema migrans, cranial nerve palsy, carditis, fatigue, and influenza-like illness	+	100%	Doxycycline, amoxicillin	Not yet available	Lane et al., 2004; de la Fuente et al., 2007
4	Helvetica spotted fever	B	*Rickettsia helvetica*	*Ixodes ricinus*	Humans, rodents	Switzerland, Laos, France, Sweden	Red spots, fever, muscle pain, headache, respiratory problems	+	Not documented	Phenoxymethylpenicillin	Not yet available	Parola and Raoult, 2001
5	Ehrlichiosis anaplasmosis/ Human granulocytic ehrlichiosis or HGE	B	*Ehrlichia chaffeensis, Ehrlichia ewingii, Ehrlichia muris-like (EML)*	*I. scapularis, I. pacificus, A. americanum (Lone Star tick)*	Humans, deer, wild and domestic dogs, domestic ruminant, rodents	US-South central, South atlantic	high fever, weakness, headache, fatigue, muscle aches (myalgia), chills	+	<1%	Doxycycline	Not yet available	Charrel et al., 2004; Yu et al., 2015
6	STARI (Southern tick-associated rash illness)	B	*Borrelia lonestari*	*A. americanum*	Humans, dogs, cats	South Central and south eastern United states	Influenza, fatigue, muscle pain, headache	+	Not documented	doxycycline	No vaccine available	Bugrysheva et al., 2001
7	Q fever	B	*C. burnetii*	*I. dentatus, I. trianguliceps, R. sanguineus, A. americanum, D. andersoni, D. reticulatus*	Goats, sheep and cattle	Worldwide spread	Clay-colored stools, chills or sweats, cough, headache, diarrhea, nausea, chest pain	+	1–11%	Doxycycline, hydroxychloroquine	Q-Vax, a whole-cell, inactivated vaccine	Sreenivasan et al., 1986; Mehla et al., 2009
8	Erythema-Migrans or rash	B	*Rickettsia helvetica*	*I. ricinus*	Humans, rodents	USA	Muscle pain, fever, headache, respiratory problems	+	Not documented	Phenoxymethylpenicillin	–	Parola and Raoult, 2001
9	Meningitis	B	*Rickettsia helvetica*	*I. ricinus*	Humans, Cats, dogs	Worldwide spread	Sudden high fever, stiff neck, seizures, headache, nausea, vomiting, sleepiness	+	10%	Penicillin G, ampicillin, chloramphenicol, cefotaxime, ceftriaxone	One polysaccharide vaccine (MPSV-4), two conjugate vaccines (MCV−4	Pavri, 1989
10	Japanese spotted fever	B	*Rickettsia japonica*	*I. ovatus*	Rodents, Humans	Japan, South Korea	Acute high fever, Headache, exanthema	+	2%	Doxycycline	No vaccine available	Ajesh et al., 2017
11	Queensland tick typhus	B	*Rickettsia australis*	*I. holocyclus*	Rodents, humans	Tasmania, Australia	Fever, muscle aches, chills, headache, and malaise	+	Not documented	Penicillin, doxycycline	No vaccine available	Carroll et al., 2010
12	Mediterranean spotted fever/Boutonneuse fever	B	*Rickettsia conorii*	*R. sanguineus*	Dogs, rodents humans	Southern Europe, southern and western asia, Africa, India	Chills, high fevers, severe headache, muscular and articular pains, and photophobia	+	2%	Tetracycline, along with chloramphenicol and quinolones	No vaccine available	Calisher et al., 1989
13	Kenya tick typhus	B	*Rickettsia conorii*	*R. sanguineus*	Humans, dogs, rodents	Kenya	High fever, headache, chills, rash, muscle pain	+	3%	Doxycycline, Chloramphenicol	No vaccine available	Dodd et al., 2011
14	Indian tick typhus	B	*Rickettsia conorii*	*R. sanguineus*	Humans, dogs, rodents	India	High fever, headache, chills, rash, muscle pain	+	1–5%	Doxycycline	No vaccine available	Muraleedharan, 2016
15	Israeli tick typhus	B	*R. conorii Israel*	*R. sanguineus*	Humans, dogs, rodents	Israel	High fever, headache, chills, rash, muscle pain	+	1-5%	Doxycycline	No vaccine available	Muraleedharan, 2016
16	Rocky Mountain spotted fever (RMSF)	B	*Rickettsia rickettsii*	*R. sanguineus, D. variabilis, D. andersoni, A. canjennense*	Humans, rodents, dogs	North, Central and South America	Fever, headache, altered mental status, myalgia, and rash	+	10–25%	Doxycycline, tetracycline	No vaccine available	Sarkar and Chatterjee, 1962
17	Astrakhan spotted fever	B	*R. conorii Astrakhan*	*R. pumilio*	Humans, rodents	North Caspian region of Russia	Fever, headache, altered mental status, myalgia, and rash	+	1–5%	Doxycycline	No vaccine available	Muraleedharan, 2016
18	Ehrlichiosis/Canine hemorrhagic fever	B	*Ehrlichia canis*	*A. americanum*	Dogs, humans	US-Southwest, Gulf coast regions	Fever, petechiae, vasculitis, lymphadenopathy, discharge from the nose and eyes, bleeding disorders, and edema of the legs and scrotum.	+	Not documented	Doxycycline, tetracycline	No vaccine available	Pattnaik, 2006
19	African tick-bite fever	B	*Rickettsia africae*	*A. hebraeum, A. Variegatum*	Humans, ruminants	Sub-Saharan Africa, West Indies	Fever, muscle ache, rash, swollen lymph nodes	+	0%	Doxycycline, chloramphenicol, and ciprofloxacin	No vaccine available	Pai and HN, 2017
20	American tick bite fever/*Rickettsia parkeri* infection	B	*Rickettsia parkeri*	*Amblyomma maculatum*	Humans, rodents	North and South America	*Fever, headache*, rash, myalgias	+	0%	Doxycycline, chloramphenicol	Not yet available	Mourya and Yadav, 2013
21	Dermacentor-borne necrosis erythema	B	*Rickettsia slovaca*	*D. marginatus*	Humans, rodents, lagomorphs	Asia, Europe	Fever, chills, rash	+	0%	Doxycycline, tetracycline	No vaccine available	Muraleedharan, 2016; Sadanandane et al., 2017
22	North Asian tick typhus/siberian tick typhus	B	*Rickettsia sibirica*	*D. nuttalli*	Rodents, humans	China, Russia, Mongolia	Rash, fever, skin ulcer at the site of tick bite, headache	+	0%	Chloramphenicol, tetracycline	No vaccine available	Patil et al., 2017
23	Lymphangitis-Associated Rickettsiosis	B	*Rickettsia mongolotimonae*	*H. asiaticum*	Rodents, humans	China, Southern France, Portugal, Africa	Rash, fever	+	0%	Doxycycline	No vaccine available	Qattan et al., 1996; Wang et al., 2009; Patil et al., 2017
24	Tick paralysis	–	Caused by toxin	*D. variabilis, D. andersoni, Ixodes holocyclus*	Humans, dogs	USA, Australia	Weakness in legs that lead to paralysis	_	10-12%	Immediate tick removal	No vaccine available	Zaki, 1997; Carletti et al., 2010
25	364D rickettsiosis	B	*Rickettsia phillipi*	*Dermacentor occidentalis*	Humans, jackrabbits, ground squirrels, and deer	California	Fever, malaise, and eschars	+	Not documented	Doxycycline	No vaccine available	Gritsun et al., 2003; Ravanini et al., 2011; Musso et al., 2015
26	Colorado tick fever	V	*Colorado tick fever virus (CTFV)*	*Dermacentor andersoni*	Humans, elks, Marmots, deer	Colorado, Idaho, Canada	Fever, chills, headaches, pain, light sensitivity, muscle pain, rash, malaise	+	0.5%	Analgesics and acetaminophen is used to relieve fever and pain, No proper treatment available	No vaccine available	Lin et al., 2003
27	Powassan disease	V	*Powassan virus*	*Ixodes cookei, Ix. marxi, Ix. spinipalpusm, Ix. scapularis, Dermacentor andersoni*, and *D. variabilis*	Humans, squirrel, mice	Eastern Russia, North America	Fever, headache, nausea, weakness	+	10%	No specific treatment is available	No vaccine available	Venugopal et al., 1994; Chávez et al., 2010
28	Tick-borne meningoencephalitis	V	*Tick-borne encephalitis virus (TBEV)*	*Ixodes scapularis, Ixodes ricinus, Ixodes persulcatus*	Rodents, humans	Europe, Russia, Asia	Fever, anorexia, muscle aches, headache, malaise, nausea, and/or vomiting	+	European subtype F.R. = <2%. Far Eastern subtype F.R. = 20−40%, Siberian subtype F.R. = 2-3%	No specific treatment is available but corticosteroids can be used as an anti-inflammatory drugs	No vaccine available	Hollidge et al., 2011; Yoshii et al., 2014
29	Crimean-Congo hemorrhagic fever	V	*Crimean-Congo hemorrhagic fever virus*	*Hyalomma marginatum, Rhipicephalusbursa*	Humans,ruminants and ostriches	Northern Africa, Southern Europe, Southern part of Asia	Flu, vomiting, black stools, hemorrhage, nosebleeds	+	10–40%	Ribavirin	Inactivated antigen CCHF vaccine	Villordo and Gamarnik, 2009; Cook, 2010
30	Louping ill	V	*Louping ill virus*	*Ixodes ricinus*	Sheep, red grouse, humans	Scotland, America	Biphasic fever, depression, ataxia, posterior paralysis, muscular incoordination, tremors, coma, and death	+	50–60%	No specific treatment is available	ATCvet	Singh and Gajadhar, 2014
31	Kyasanur Forest Disease (KFD)	V	*Kyasanur Forest Disease Virus*	*Haemaphysalis spinigera*	Rats, squirrels, mice, shrews, porcupines, humans	South Asia	High fever, hemorrhage, headache, bleeding from gums, nasal cavity throat, and gastro intestinal bleeding	+	3–10%	No specific treatment is available	Formalin-inactivated KFDV	Walsh et al., 1993; Jones et al., 2013
32	Alkhurma hemorrhagic fever (AHFV)	V	*Alkhurma virus*	*Ornithodoros savignyi, Hyalomma dromedari*	Camel, sheep	Saudi Arabia, Egypt	Non-specific flu-like symptoms, fever, anorexia, general malaise, diarrhea, vomiting, neurologic, and hemorrhagic symptoms	+	Above 30%	No specific treatment is available	No vaccine available	Charrel et al., 2007; Carletti et al., 2010
33	Heartland Virus Disease	V	*Heartland Virus*	*Lone Star tick (Amblyomma americanum)*	White-tailed deer, raccoon	U.S.	Lethargy headaches, myalgia, loss of appetite, nausea, diarrhea, weight loss, arthralgia, leukopenia and thrombocytopenia	+	12%	No specific treatment is available	No vaccine available	McMullan et al., 2012; Angela et al., 2016
34	Bourbon virus disease	V	*Bourbon viruses*	*Unknown*	Unknown	Midwest and southern United States	Fever, tiredness, rash, headache, other body aches, nausea, and vomiting, thrombocytopenia and leukopenia	+	−	No specific treatment is available	No vaccine available	Devi, 2015; Petersen et al., 2016
35	Cytauxzoonosis	P	*C. felis*	*D. variabilis*	Bobcat, cougar	South east, South USA	Inappetance, lethargy, high fever, respiratory distress, tachycardia	–	Without treatment = 97%, with treatment = 40%	Imidocarb dipropionate and a combination of azithromycin and atovaquone	No vaccine available	Singh et al., 2011; Gritsun et al., 2014; Saini et al., 2016
36	Babesiosis	P	*Babesia microti*	*I. scapularis, I. pacificus*	Rodents, humans	US-Northeast, West coast	Fever, chills, sweats, nausea, or fatigue headache, body aches, loss of appetite	+	42%	Combination of clindamycin, doxycycline, and azithromycin	No vaccine available	de la Fuente et al., 2017

Though many tick control chemical options like use of organophosphates or pyrethroids are available, the number of TBDs associated with domestic and wild animals is increasing even in the developed countries of Europe, where 50,000 cases are reported annually in humans (Sonenshine et al., 2006). However, there is a significant need for very practical and comprehensive approach toward the better management of these TBDs. Along with chemical control, biological control methods by the assistance of natural predators like spiders, beetles, and nematodes can be another alternative approach based on the integrated pest management (Benjamin et al., 2002). Due to remarkable development in molecular biology, advancements have been made in tick borne pathogens diagnosis; however, still there is a need for continuous organized research to analyze and comprehend diversity of tick borne pathogens in different geographical regions of the world (Charrel et al., 2004).

Kyasanur Forest Disease is one of tick-borne associated diseases of flaviviruses caused by infected tick bite of *Haemaphysalis spingera*. It was first discovered in 1957 in India in Kyasanar forest of Shimoga district, state of Karnataka. Kyasanur Forest Disease Virus (KFDV) falls in mammalian tickborne virus group, belonging to family *Flaviviridae*, genus *Flavivirus* (Yadav et al., 2014). It is principally transmitted to humans and animals by tick vector *Haemaphysalis spinigera*. Symptoms of this disease include but not limited to a frontal headache, severe prostration, high fever and conjunctivitis, bleeding from nose, mouth, and gastrointestinal tract. The incubation period of this KFDV is ~3–8 days (Work et al., 1957).

Kyasanur Forest Disease Virus is classified in risk group 4 pathogens and causes endemic disease whose ecology and epidemiology is unpredictable because it is a forest borne disease and involves different vertebrate species (including monkeys, shrews, bats, birds, and small rodents) for its transmission cycle (Carroll et al., 2010).

The current review significantly emphasizes the importance of limiting KFDV spread due to its severe pathogenicity, explores pathogenicity pathway, epidemiology of KFDV, transmission, prevalence, clinical manifestation, and highlights current, and future perspectives of research on this disease. Tick-borne diseases caused by possible causative agents (i.e., bacteria, viruses, and protozoa) have been rigorously reviewed, in tabular form (Table 1), with reference to geographical distribution, symptoms, zoonosis, fatality rate, availability of therapeutics, and vaccination. Finally, treatment, clinical management, and prophylactic measures of KFDV are discussed.

## Etiology and geographical distribution

The etiologic agent of Kyasanur Forest Disease is a virus i.e., KFDV, which belongs to tick-borne encephalitis serocomplex of flaviviruses that also includes tick-borne encephalitis, Omsk hemorrhagic fever, Langat, Powassan, and Louping-ill viruses (Calisher et al., 1989). The spherical virus (40–65 nm size) is enveloped with an icosahedral nucleocapsid and consists of a single stranded RNA genome of positive polarity that is 10,774 bases in length and codes for a single polyprotein: C-prM-E-NS1-NS2A-NS2B-NS3-NS4A-NS4B-NS5 (Dodd et al., 2011). It is, however, an epidemiologically understudied virus.

**Figure 1:**
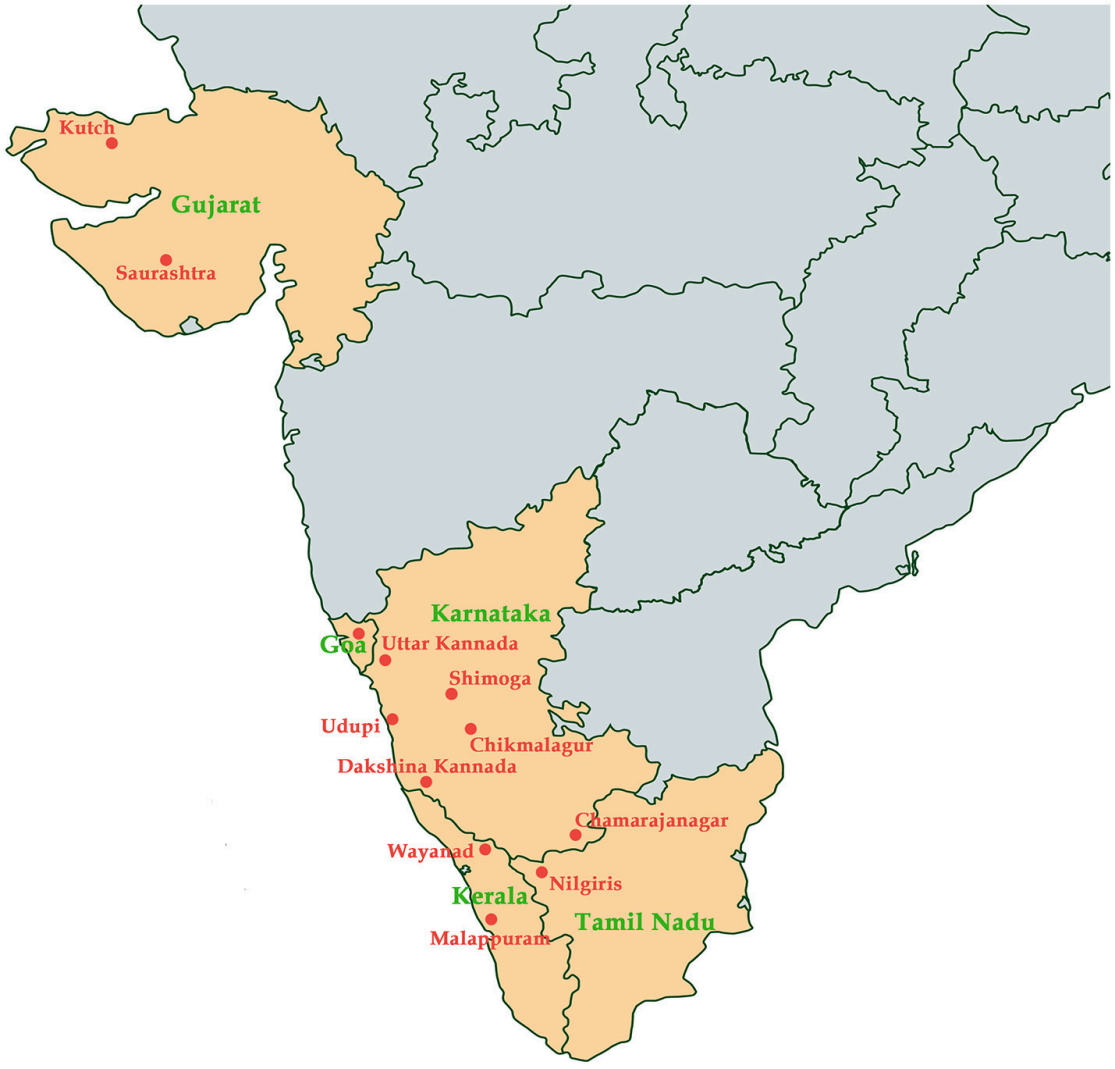
Map indicating states (colored in orange) in India and the regions (labeled in red) where Kyasanur Forest Disease has been reported.

KFD was initially reported in Kyasanaur forest of Shimoga district in India. Numerous deaths of black-faced langur (*Semnopithecus entellus*) and red-faced bonnet monkeys (*Macaca radiata*) were observed. In addition, the disease also affected the humans who had visited the affected forest at that time (Mehla et al., 2009). Over the past 5 decades, about 400-500 human cases have been reported annually. The tick species *Haemaphysalis spinginera* is widely distributed in the deciduous and evergreen forests of India and Sri Lanka (Sreenivasan et al., 1986). KFD was reported to be endemic to Sagar, Sorab, and Shikarpur Taluks of district Shimoga. During 1957–1972, various virus isolates were obtained from Karnataka and were retained in the depository of National Institute of Virology in Pune, India (Muraleedharan, 2016). By 1964–1965, the death of monkeys was reported only in the previously known affected areas and by 1965–1966, the endemic was extended toward the south-east forests of Sagar town covering approximately 30 square km. By 1966–1969 the epizootics appeared in the north-west of Sorab town and by the end of 1973, it extended to distant places away from the initial hotspots. Antibodies against KFDV were detected in the people living in Kutch and Saurashtra of Gujrat state in India, around 1,200 km away from Karnataka state which was the main focus of KFD (Sarkar and Chatterjee, 1962). Since 1957, after the discovery of KFDV many sporadic cases have been observed in the endemic state of Karnataka every year, mostly in five major districts; Shimoga, Chikmagalur, Udupi, Uttar Kannada, and Dakshina Kannada (Pattnaik, 2006). From the year 2004–2012, many outbreaks of KFD were reported with accumulated 556 human cases in four districts of Karnataka state (Pai and HN, 2017). From 2012 to 2013, KFD outbreak was reported in the Bandipur Tiger Reserve in Chamarajanagar among the forest workers. During the same period, the virus was detected in ticks and/or monkeys in Nilgiri and Wayanad (Mourya and Yadav, 2013). During 2014–2015, KFD outbreaks were explicitly observed in new regions of Wayanad and Malappuram districts of Kerala (Sadanandane et al., 2017); and recently, KFD activity has been reported in Goa, India (Patil et al., 2017). Spread of KFDV in various regions in India has been documented in Figure 1.

Previous literature on the current area of interest has described identification of KFDV in Chinese population and KFDV variants in Saudi population (Qattan et al., 1996; Wang et al., 2009). Viruses isolated from patients with hemorrhagic fever were identified as KFDV variants in Makkah region of Saudi Arabia and named Alkhurma hemorrhagic fever virus (AHFV). Despite being closely related on genetic level, KFDV and AHFV are strikingly different in their mode of pathogenesis and tissue tropism (Sawatsky et al., 2014). However, the KFDV isolate reported in 1989 from Chinese region was revealed to be unauthentic by Mehla et al. (2009). Though the two strains were reported 32 years and about 3,000 km apart, the unidentified isolate exhibited a difference of only 1 nt [1/1,320(0.08%)] from the reference KFDV strain (P9605), which was identified in Indian subcontinent and distributed across the globe to arbovirus reference laboratories (Mehla et al., 2009).

## Molecular epidemiology

**Figure 2:**
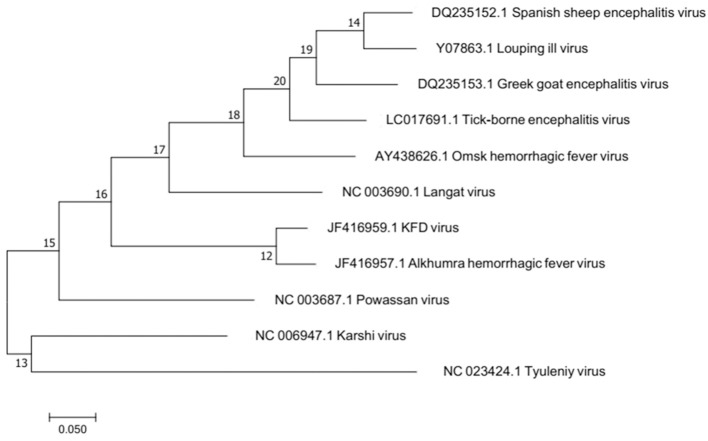
Molecular phylogenetic analysis by Maximum Likelihood method. The evolutionary history was inferred by using the Maximum Likelihood method based on the Tamura-Nei model. The tree with the highest log likelihood is shown. Alkumra hemorrhagic fever virus can be seen as the most closely related neighbor to KFD in the phylogenetic tree.

Molecular epidemiological studies have contributed to unravel variants of KFDV. AHFV is located in Saudi Arabia and Egypt (Zaki, 1997; Carletti et al., 2010; Ravanini et al., 2011; Musso et al., 2015). It was isolated during 1994–1995 from hemorrhagic fever patients in Makkah, Saudi Arabia (Zaki, 1997). Sequence identity of KFDV as revealed by Blast results is 97% with Alkhumra virus, 79.6% with TBEV, 79% with Louping ill virus, 78.7% with Omsk, 78.7% with Langat virus, and 76.3% with Powassan virus. Phylogenetic relationship of these viruses has been depicted in the phylogenetic tree based on maximum likelihood is illustrated in Figure 2. Omsk Hemorrhagic fever virus (OHFV) that is prevalent in Western Siberia, Russia, has also been found to be distantly related to KFDV. KFDV, AHFV, and OHFV are unique members of TBE serocomplex as they cause hemorrhagic fever with neurological implications (Gritsun et al., 2003). During the past few millennia, tick-borne flaviviruses have evolved much and were dispersed from north to west across European and Asian forests. Bayesian coalescence analysis (based on partial E and NS5 sequences) of Indian KFDV isolates and variants from China and Saudi Arabia revealed a mean evolutionary rate of ≈6.4 × 10^−4^ substitutions/site/year for KFDV while a recent analysis based on full-length sequences (AHFV n = 18 and KFDV n = 3) revealed a much slower evolutionary rate i.e., (9.2 × 10^−5^ substitutions/site/year) and thus, is suggestive of a much ancient ancestry of KFDV (Mehla et al., 2009). Analysis by Mehla et al. (2009) showed that isolates from India and Saudi Arabia share common ancestry despite their wide geographic distribution. KFD and ALK viruses, on the basis of phylogeny, form a clade within the TBE serocomplex that is distinct from OHF and other viruses. However, an analysis of the phylogenetic position of KFDV/AHFV and OHFV does not uncover a genetic lineage-linked to their hemorrhagic disease producing ability (Lin et al., 2003).

## Viral determinants of pathogenesis

Despite its high pathogenicity and epidemiological significance, there have been very few detailed molecular studies on KFD and this remains a reason that the genetic basis of disease produced by this flavivirus is not yet understood in complete detail; only partial sequence of the virus was characterized by Kuno et al. (1998). Grard et al. (2007) performed analysis of complete coding sequences of tick-borne flaviviruses including KFDV and Dodd et al. (2011) sequenced three full-length KFDV genomes using PCR based amplification and sequencing technology for evolutionary analysis of AHFV and KFDV. The entire genome of KFDV was sequenced by Cook et al. (2012) in fragments and full-length cDNA was constructed using reverse genetics system, step-by-step, to build the complete genome (10,774 nucleotides) of KFDV.

The ability of KFDV, AHFV, and OHFV to produce hemorrhagic disease suggests the existence of molecular determinants, within the genome, associated with pathogenicity of these viruses. Among the structural proteins, E protein of these flaviviruses is a critical causal element of tissue tropism (Lin et al., 2003). Mediating the entry of the virus into the host cell, this protein is responsible for immunogenic and phenotypic properties of the virus, thereby having significant roles in pathogenesis and immune evasion (Barker et al., 2009).

A comparison of amino acid sequences of KFDV E protein has revealed a similarity of 77.4–81.3% to tick-borne flaviviruses (Venugopal et al., 1994). Hemorrhagic fever producing tick-borne viruses have three similar amino acid residues in the E protein i.e., E- 457, E- 489 and E- 76. In particular, an alanine (Ala) residue at position 76 instead of threonine eliminates hydrogen bonding potential and may cause destabilization in the E protein, thereby increasing the chances of “accidental” fusion events of hemorrhagic fever viruses (Lin et al., 2003). Also, Asn- 67 is highly favored in hemorrhagic fever viruses (Barker et al., 2009).

An analysis of new KFDV and OHFV sequences has identified a (T → A) mutation at E- 76 which may be of particular importance in inducing hemorrhagic presentation. Various specific motifs present in KFDV E protein are associated with hemorrhagic manifestations such as the AKG motif (Vir C positions 2– 4), a basic residue at position Vir C- 9, EGSK motif (Lin et al., 2003), EHLPTA marker with a T → K substitution and a unique AQE marker at amino acid positions 232–234 (Venugopal et al., 1994). Also, the domain III of E protein comprises of epitopes that are useful for flavivirus serologic diagnosis and as immunization targets (Chávez et al., 2010).

Among the nonstructural proteins of flaviviruses, NS1 and NS5 have been implicated as important determinants of disease. Only NS1 glycoprotein has been found on infected cell surfaces. Association of NS1 with intracellular membranes along with co-localization with viral dsRNA replicative form suggests its role in viral replication (Bugrysheva et al., 2001). NS5 also confers replication advantage to KFDV by being the primary IFN antagonist. It is a highly conserved protein among flaviviruses, with 80–90% similarity among the TBE virus serogroup (Hollidge et al., 2011). Four amino acids in the proximity of C-terminus of the NS5 protein have been determined to be principally accountable for the development of neurological disease in mice following TBEV infection as mutation of these amino acids did not impact viral replication or histopathological features (Yoshii et al., 2014).

The 5 ′ and 3′ UTRs flanking the coding region of flaviviruses also influence viral replication. Stem-loop structures (secondary structures) in these UTR regions are utilized by KFDV and other flaviviruses to assist in their genomic replication and translation (Villordo and Gamarnik, 2009). Two 5′stem loops (5′ SL) and five 3′ SL were determined in KFDV genome and were observed to be similar to those of TBEV-Neudoerfl strain; the 5′ UTR regions being 70.8% similar (Cook, 2010). Duplicated RNA structures present in the 3′ UTR of flaviviruses are imperative either as enhancers of viral replication and/or antagonists of antiviral innate immunity of cells, the latter determining the virus host range as well as its pathogenetic characteristics (Gritsun et al., 2014). One of the groups scanned KFDV virus genome and predicted eight mature miRNAs that regulate two host target genes i.e., ANGPT1 (angiopoietin 1) and TFRC (transferrin receptor) which are involved in hemorrhagic fever and neurological manifestations (Saini et al., 2016).

## Potential ecological drivers of KFD prevalence

Tick-borne KFD has emanated as a grave health problem in South Asia, causing significant morbidity and mortality. Various ecological and anthropogenic changes have driven the emergence and amplification of tick-borne KFDV. Factors such as deforestation and land use practices, climate change, diverse migratory bird population, population densities, decreasing wildlife habitats and changes in human behavior have been speculated to play a vital role in the resurgence of this zoonotic pathogen at a higher frequency in endemic regions and its emergence in newer areas (Singh and Gajadhar, 2014). This is because anthropogenic environmental changes alter population structure and migration of wildlife and decrease biodiversity by the creation of environments that support distinct hosts, vectors and/or pathogen (Jones et al., 2013).

Deforestation has been reported as a major reason for the transmission of KFD. An immense loss of forest cover in the Western Ghats mountain ranges, where the virus is geographically distributed, has occurred due to agricultural intensification and increased human activities, thereby enhancing the risk of disease emergence in new zones (Jha et al., 2000). Removal of trees results in a quick occupation of shrubs that offers a conducive environment for rodents and birds which subsequently acts as hosts for the growing larvae and nymphs (Walsh et al., 1993). The microclimate within the bushes and under leaves serves significant roles for tick vectors as affirmed by seroprevalence studies in various wild animals and by experimentally induced infections in the laboratory (Pattnaik, 2006). Increased agricultural activity and encroachment of the virus into Indian forests and/or previously unutilized lands by primate species and highly mobile hosts such as bats and birds play a potential role in increasing contacts between humans, domestic, and wild animals; thus causing dispersion of the ticks and tick-borne pathogenic virus (Isabirye-Basuta and Lwanga, 2008; Hasle et al., 2009; Weaver and Reisen, 2010). The introduction of this novel virus into human populations in 1957 and the resulting occurrence of seasonal outbreaks demonstrate a chief role of wildlife in the emergence of this zoonotic pathogen (Singh and Gajadhar, 2014). Transmission by animal migration or bats might have been the cause of disease emergence in the new KFDV zones-Sattari taluk of Goa, Sindhudurg district of Maharashtra State, Kerala and Tamil Nadu (Ajesh et al., 2017).

**Figure 3:**
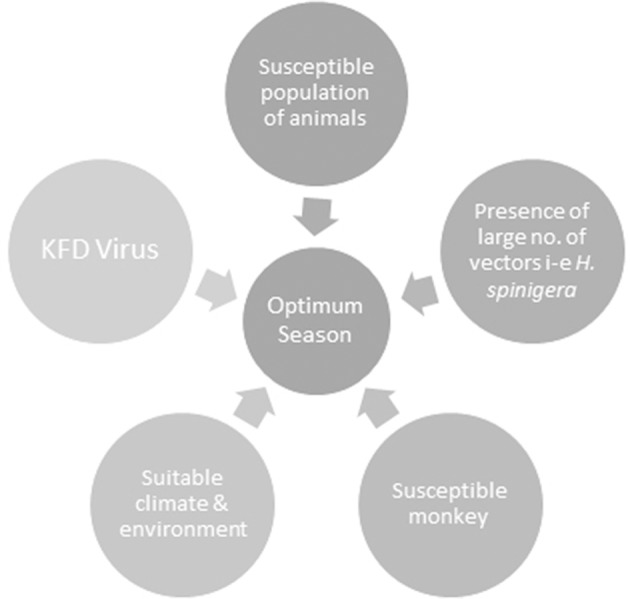
The confluence of factors responsible for Kyasanur Forest disease (KFD). All of these factors when present in right proportions result in an optimum season for the development of KFD.

Climate change as a result of deforestation also exerts a transient impact on KFD epidemics by increasing hosts, reservoirs and vector base. Climate is a critical element of temporal and geographic distribution of ticks, their life cycle, the evolution of arboviruses and transmission to vertebrate hosts (Ogden et al., 2005). KFDV that harbors in ticks and circulates among wild animals is vulnerable to climate change. Climatic variability can cause an alteration in the extent of the area that supports wildlife as well as leading to an expansion of existing peripheries of agricultural activities, thus increasing the possibility of contact between previously non-interacted species and leading to KFD spread (Hoeve et al., 1988). Such changes have been speculated to increase the risk of KFD in India. Destruction of forest cover in the Western Ghats caused significant changes in annual precipitation levels in the region that will possibly affect patterns of this wildlife disease via survival of vectors or by influencing host-parasite relationships (Ajesh et al., 2017). Prevalence of vector-borne zoonoses is probable to escalate in the near future due to the impacts of global warming in India (Karunamoorthi, 2012). The occurrence of a marked number of cases in Kerala's Wayanad district over the last 4 years evinces the permanent establishment of the infection in new regions and its continued growing threat to humans upon exposure. Further research is required to decipher the potential of the KFDV in invading new geographic regions and subsequently creating significant public and veterinary health problems (Ajesh et al., 2017). The confluence of factors responsible for KFD is shown in Figure 3.

## Transmission of KFDV

KFD is a zoonotic disease, in which humans play no major role in the infection transmission but are secondary hosts. Ticks serve as vectors and main reservoirs of KFDV (Sadanandane et al., 2017). In the natural transmission cycle of KFD, the *Haemophysalis* ticks transmit the infection to a vertebrate host that is non-human such as bird or mammal (Ajesh et al., 2017). *H. turturis* and *H. spinigera* are the two main vector species of KFDV as a number of isolations of KFDV have been procured from these two species. In a study conducted in Malappuram and Wayanad districts of Kerala, India, in 2015, *H. turturis* and *H. spinigera* accounted for about >40% of the entire ticks specimens that were collected from the Karulai and kurichiyat forest areas (Sadanandane et al., 2017). Other species acting as vectors in transmission include *H. formosensis, H. papuanakinneari, H. cuspidata, H. bispinosa, H. aculeate, H. kyasanurensis and H. wellingtoni* (Work et al., 1957). However, some other genera that are reported for their capability of infection development are *Dermacentor, Argas, Rhipicephalus, Hyalomma, Ixodes, and Ornithodoros* (Ajesh et al., 2017).

The major vector of the Kyasanur Forest Disease *H. spinigera* is prevalent in the state Karnataka of India (Geevarghese and Mishra, 2011). From the huge number of isolations that are obtained from various ticks, around 95% are contributed by *H. spinigera*, which is one of the main species of ticks located in the jungle (Varma et al., 1960).

**Figure 4:**
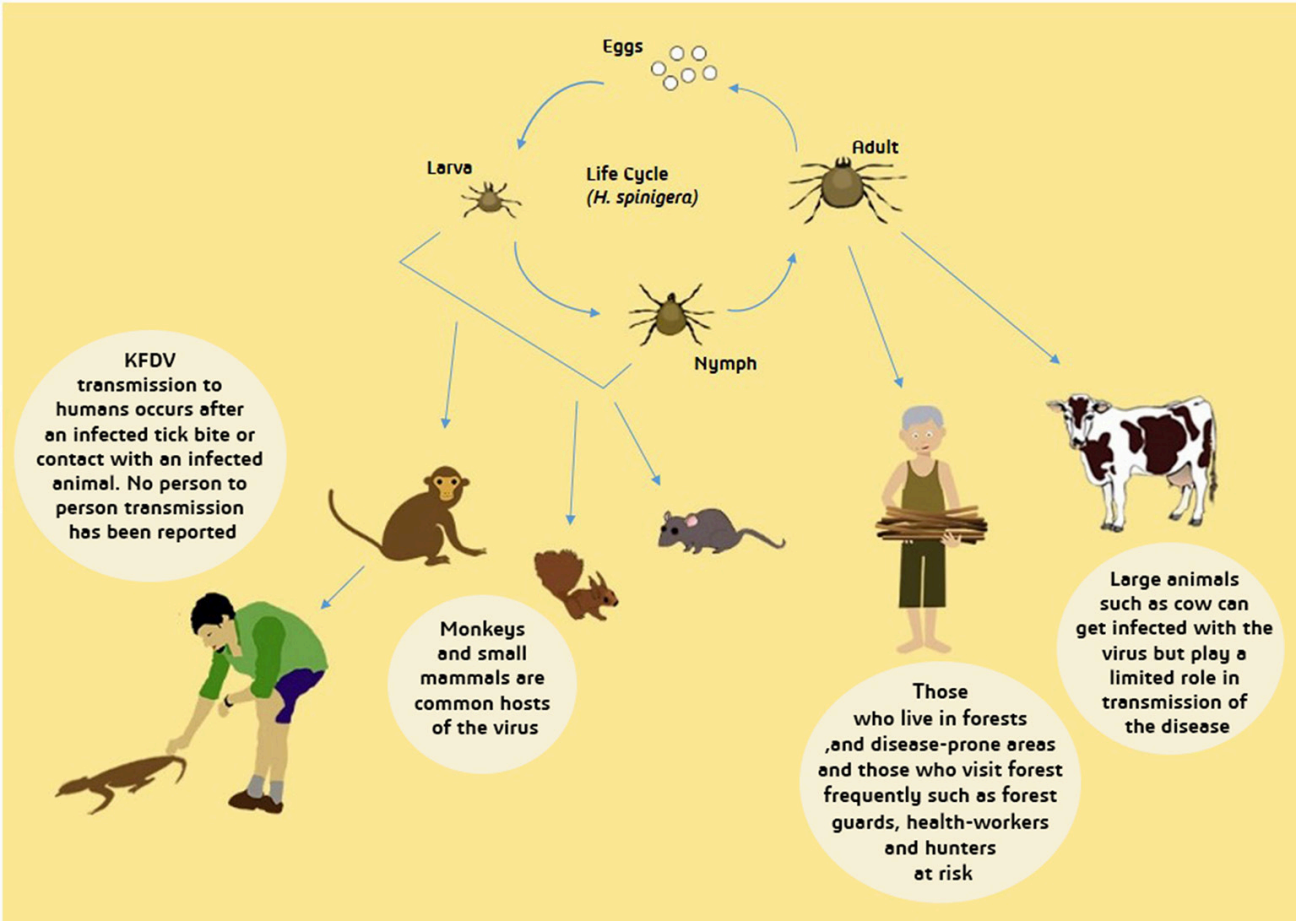
Transmission of Kyasanur Forest Disease Virus (KFDV). From tick larva and nymph, transmission to primary hosts- small mammals and monkeys- occur and afterwards to humans who experience contact with dead animal of infected tick bite. Infected adult tick can directly transmit the virus to humans and large animals. Transmission from infected large animals is limited while human-human transmission has not yet been reported. People inhabiting forests are more likely to be infected with KFDV.

Ixodes species of ticks is also known to be one of the main disease vectors of humans and animals, therefore, it also acts as an important KFD reservoir (Boshell and Rajagopalan, 1968a). Ticks are able to develop an infection in any phase of their life cycle. Through the transstadial form of transmission, KFDV is delivered to subsequent stages of ticks and is also transovarially transmitted to the mature tick progeny (Ajesh et al., 2017). Another form of transmission- that is the most likely and a more efficient route of transmission of KFDV- is through co-feeding of ticks on a mammal (host) which enables viral transmission between ticks without host infection (Randolph, 2011; Mansfield et al., 2017). Figure 4 depicts wide-ranging hosts of KFDV which include humans, species of ticks, rodents (forest rats, shrews, white-bellied rat and white-tailed rat), bats, squirrels, ground-dwelling birds, Indian crested porcupines and monkeys (black–faced langur, bonnet macaque, and gray langur).

Reservoirs are extremely important for the existence of several viruses. The best upkeep hosts are rodents as their generation period is very short, they constantly present a new group of animals (Boshell and Rajagopalan, 1968b). In the enzootic regions, KFDV is circulated and maintained in little mammals particularly shrews, rodents, ticks and ground birds (Mourya and Yadav, 2013). Moreover, the red-faced bonnet monkeys and black-faced langurs are also extremely susceptible to the KFDV (Sreenivasan et al., 1986). The wild monkeys are infected through an infected tick bite and then followed by the spread of KFDV among the population. The infected ticks fall from the infected monkeys carcasses when the monkeys die, thereby generating new hot-spots of infected ticks. Thus visiting such areas in woodlands and forests is dangerous and chances of exposure to the infected ticks are very high (Sadanandane et al., 2017).

Changes in ecosystem caused by human intrusion could direct a mode for the transmission of KFDV from wild animals to humans (John et al., 2014). Walsh et al. (1993) documented cutting down of trees as one of the main cause for the distribution of Kyasanur Forest Disease. A number of cases have been reported in the nearby areas of forest from Indian states like Karnataka, Tamil, Nadu, Goa and Kerala (Kasabi et al., 2013b; Tandale et al., 2015; Mourya and Yadav, 2016; Patil et al., 2017; Sadanandane et al., 2017) where the spread occurrence of tick vector *Haemophysalis* is very wide-ranging. Due to close interaction with the local animals of the affected jungle in these particular regions, the chances of transporting the infested ticks to nearby rural areas are escalated and hence the possibility of the infected tick bites to the people of that area is also enhanced (Murhekar et al., 2015).

Ticks infect and spread the infection to animals mainly and humans are dead-end hosts of KFDV as it is less likely for infection to be transmitted from human to other hosts (Mourya and Yadav, 2013). Humans become infected by bites from infected nymphs (Sadanandane et al., 2017). Unfed nymphs transmit the infection to humans and are extremely anthropophilic, preferring human beings than other animals (Pattnaik, 2006). It is not known whether the intracellular machinery of asymptomatic hosts can support viral replication and formation of infectious virions, and how the humans might shed the virus for transmission between humans.

After the infected nymphs mature to adults, female ticks lay eggs that are hatched to produce larvae, which further infect monkeys and small mammals, also infecting humans accidentally, and keeping feeding on their hosts. Later, the larvae grow and develop into nymphs and in this way, the life cycles go over again (Mourya et al., 2014). Figure 4 depicts the life cycle of ticks and transmission of KFDV from ticks to animals and humans.

## Clinical manifestations of KFD

KFD is an acute, biphasic illness in humans, composed of acute and convalescent phases. A presymptomatic or an incubation period of 2–7 days occurs after a tick bite. It is followed by an abrupt onset of illness that lasts for about 1–2 weeks. This febrile period includes clinical symptoms such as sudden chills, frontal headache, vomiting, diarrhea, sore throat, high fever (104°F), generalized muscle pain and a severe degree of prostration (Liu, 2014; Mourya et al., 2014). In some patients, one important diagnostic sign is the papulovesicular eruption on the soft palate i.e., blisters on the upper, inner mouth; however, there have been no reports of skin eruption (Shiji et al., 2016). Hemorrhagic manifestations such as epistaxis, hemoptysis, bleeding from the gums and gastrointestinal bleeding are observed during this viremic phase. Haematemesis or the appearance of fresh blood in the stool may also be observed (Chandran et al., 2016). Other common symptoms include insomnia, decreased heart rate and blood pressure and extremities. Ophthalmic presentation of this disease includes hemorrhages in the conjunctiva, vitreous humor, and retina, mild iritis, the opacity of lens and keratitis (Grard et al., 2007). The chief pathologic features include hemorrhagic pneumonitis, hepatomegaly with parenchymatic degeneration, nephrosis, characteristic reticulo-endothelial cells in spleen and liver along with increased erythrophagocytosis in spleen (Pattnaik, 2006). Hematologic abnormalities are also observed in KFD. These include leucopenia (reduction in white blood cell count), thrombocytopenia (reduction in platelet count) and reduced number of red blood cells (Pavri, 1989; Shiji et al., 2016). Elevated levels of liver enzymes and anemia have also been seen in patients (Pattnaik, 2006).

The acute viremic phase of the disease is followed by a prolonged convalescent phase that constitutes recovery and it may be up to 4 weeks. However, about 10-20% of patients experience a recurrence of symptoms after 1–2 weeks of the first febrile stage (Jayarajan, 2014). This second non-viremic phase, lasting for about 2–12 days, is characterized by the same symptoms observed in the first phase along with neurological complications. Encephalitic features such as mental disturbance, giddiness, stiff neck, abnormality of reflexes, confusion, and tremors have been reported in 35% of affected individuals whereas another 55% of patients suffering the second wave of symptoms of KFD may succumb to bradycardia, meningoencephalitis, hemorrhagic fever manifestations (profuse gastrointestinal bleeding, bronchopneumonia), conjunctival inflammation, coma and possibly death (Nichter, 1987; Pattnaik, 2006; Chandran et al., 2016). Although details of pathobiology differ with the characteristic virus but bleeding in most hemorrhagic fevers has been reported to be due to disseminated intravascular coagulation (DIC) that causes depletion of coagulation factors to induce massive plasma leakage and hypovolemia, leading to multi organ failure and shock, and, in few cases, even death (Khan et al., 2008). Bonnet monkeys and langurs exhibit the same disease symptoms as humans but with a much higher mortality rate i.e., about 85% (Dobler, 2010).

## Proposed mechanism of KFDV pathogenesis

**Figure 5:**
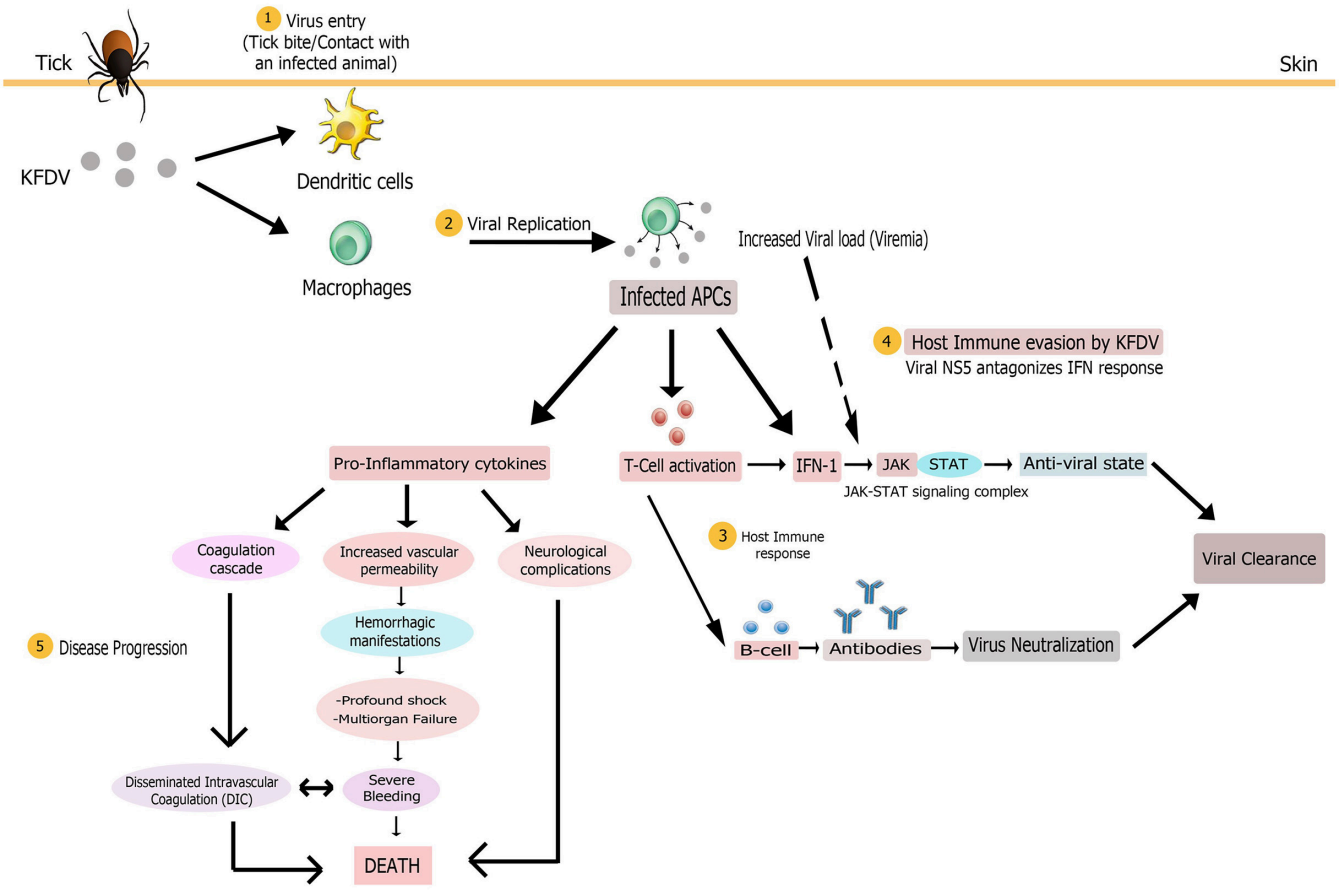
Proposed pathogenesis model of Kyasanur Forest Disease. Virus enters (1) in body on tick bite or through contact with an infected animal. Virus initially targets macrophages and dendritic cells. Multiplication of virus (2) in these host cells yields high viremia, leading to systemic spread of the virus to spleen, liver, and other replication sites to produce disease symptoms. The infected antigen presenting cells (APCs), that present viral antigens to T cells, could release large amounts of pro-inflammatory cytokines early after infection and also modulate host immune response (3) via type 1 interferon production. Antigen positive (activated) T cells could also produce IFN-1. Subsequent activation of the JAK-STAT signaling induces an antiviral state for alleviating virus burden. Humoral immune response via production of antibodies by activated B cells might also assist in viral clearance from the body. To counter the host immune response, KFDV employs its NS5 non-structural protein to antagonize IFN response (4) by inhibiting JAK-STAT pathway, possibly bringing about uncontrolled viral replication and poor immune response. The multi-systemic illness might be attributed to the pro-inflammatory cytokine storm that could contribute to immunosuppression and disease progression (5) by inducing disseminated intravascular coagulation (DIC), neurological complications and vascular dysfunction that leads to hemorrhagic manifestations, multi-organ failure and shock. These complications altogether finally result in death.

The interplay of various pathologic mechanisms leading to this multisystemic illness in monkeys and humans is complicated. Some clinical and laboratory findings have helped to unravel the pathobiology of KFD but it is still not completely known due to the lack of suitable *in vivo* and *in vitro* models of disease which has led to considerably low advances in the understanding of KFDV-induced pathogenesis. Nevertheless, some mechanisms underlying pathogenesis are common to all flaviviruses (Pastorino et al., 2010). Based on the published knowledge of immunopathology of KFDV and closely related tick borne flavivirus and/or hemorrhagic fever viruses, a hypothesized elucidation of the pathogenesis of KFDV infection has been presented in this section (Figure 5).

Transmission of KFDV to a vertebrate host probably occurs either via contact with an infected animal or a tick bite that injects the virus and saliva components into the skin site of feeding (Nuttall et al., 1994). The virus, like other flaviviruses, then infects its target cells, possibly monocytes and/or macrophages and dendritic cells. Based on the general mechanism of flaviviral replication, it is speculated that mediated by the E protein, the virus adheres to the cell surface and enters the target cell by receptor-mediated endocytosis. It then exploits the host's cytoplasmic machinery to enable viral multiplication for the initiation of infection. The positive-stranded viral RNA is translated into three structural and seven non-structural proteins that are imperative for RNA replication and polyprotein processing. Following viral replication, pathogenic virions are released from target cells by exocytosis (Mackenzie, 2005; Shi, 2012), ultimately causing high titer viremia and symptoms of the disease. A large amount of virus release from these antigen presenting cells (i.e., macrophages and dendritic cells) early after infection modifies their antigen-presenting and cytokine-producing functions that presumably contributes to pathogenesis noticed in most of the viral hemorrhagic fevers (VHFs), including Ebola and Lassa viruses (Mahanty et al., 2003). Dysregulated proinflammatory cytokine release from these cells could promote immunosuppression and increased vascular permeability, bringing about hemorrhagic signs (as observed in Ebola virus infection Baize et al., 2002) and other clinical manifestations characteristic of KFD. The exact cytokine profile associated with the immunopathogenesis of KFDV infection is as yet unclear. The infected APCs may also act as carriers to transport the virus to its other replication sites such as spleen, liver and other organs to give rise to pantropism of KFDV and, virtually, of all hemorrhagic fever viruses such as Rift valley fever virus (McElroy and Nichol, 2012). Also, platelets are implicated in VHFs (Zapata et al., 2014) but platelet-KFDV interactions in KFD pathogenesis are not understood.

Host immune response occurring in KFDV infection has not been investigated and thus its protective and/or pathological role cannot be characterized in simple terms. However, studies have shown that detection of flavivirus components in the cell triggers cytoplasmic signaling cascades, resulting in type 1 IFN production (IFN-α and IFN-β) by infected cells. Subsequent activation of the JAK/STAT signaling induces an antiviral state for the alleviation of virus burden and initiation of an adaptive immune response (de la Fuente et al., 2017). The antiviral state also allows expression of IFN-stimulated genes (ISG) that confer protection to naïve cells from infection (Randall and Goodbourn, 2008). Since interferons play a role in controlling viral replication, their rapid production is an important component of innate immune signaling for the clearance of viral infection (Hollidge et al., 2011). The host immune system also produces antibodies for the elimination of KFDV. In the first week after the KFD onsets, complement fixing (CF) and haemagglutination inhibition (HI) antibodies begin to rise while neutralizing antibodies are produced during the second week, and the titers reach the peak in the third to the fourth week. These antibodies recognize the epitopes present chiefly in the flaviviral E glycoprotein; and thus prevent viral attachment, internalization, and replication within cells. Antibody-mediated immunity to KFDV has been observed to persist for a decade even in the absence of recurrence of disease (Achar et al., 1981). However, IgE has been observed as a cofactor in the immunopathology of KFD (Pavri, 1989).

To counter the host immunological response, KFDV (like other flaviviruses) employs antagonistic interactions for immunosuppression of host responses to infection (Pastorino et al., 2010). It is not clearly known how KFDV interferes with immunity of its host after the establishment of infection. However, it has been hypothesized that severity of the disease associated with viral infection corresponds with the potential of the flavivirus to thwart the IFN response (Hollidge et al., 2011). KFDV employs its NS5 non-structural protein to antagonize IFN response by inhibiting JAK-STAT signaling since this pathway is crucial in limiting viral replication. Thus, this protein might have a pivotal role in KFD pathogenesis by allowing the virus to take over cells (Cook et al., 2012; Best, 2017). The anti-JAK/STAT pathway function of NS5 of KFDV is due to its RNA-dependent RNA polymerase (RdRp) domain (Cook et al., 2012). Experimental evidence has demonstrated the ineffectiveness of different concentrations of IFN-α/β subtypes against KFDV replication whereby virus titers increased by 102.1 TCID50/mL and KFD infection was not reduced by 90% using either of the IFN subtypes, hence emphasizing the role of NS5 in viral replication. Though NS5 can obstruct IFN signaling, its duration, however, has not been investigated (Cook et al., 2016). Thus, the final balance between immune protection and immunopathology substantially governs the consequence of viral infection and disease severity (Robertson et al., 2009).

## Animal models of KFD

According to various studies, several animal models like squirrels, porcupines, shrews, rats, bonnet macaques, and mice have been infected with KFDV to study its pathogenesis (Iyer et al., 1960; Bhat et al., 1976; Kenyon et al., 1992). Disease symptoms were observed in rodents, squirrels and bonnet macaque, and pathological studies were only carried out in bonnet macaques and mice.

### Non-human primates (NHPs) model

Among NHPs, species *Bonnet macaque* (*M. radiata)* is specifically susceptible to KFDV infection. These animals experimentally infected with KFDV were reported to develop anemia, hypotension, thrombocytopenia, diarrhea, leucopenia, elevated liver enzymes level, encephalitis and visceral organs disease (Kenyon et al., 1992).

KFDV infection in macaques causes hematological changes including abnormally low lymphocytes level and anemia and histopathlogical changes in GI tract and lymphoid organs. The initial study detected lymphoid hyperplasia, a condition characterized by non-specific necrosis within liver and kidney, and disseminated non-supportive encephalomyelitis. Further investigations discovered that bonnet macaques infected with KFDV develop infection lead to alterations in fatty deposition in the liver, resulting in depletion of lymphocytes along with occasional necrosis of lymphoid organs, as well as loss of GI tract architecture without any evidence of neurologic involvement (Iyer et al., 1960; Webb and Burston, 1966).

Among various KFDV-infected macaques, peripheral and visceral lymph nodes, spleens, and all mucosal lymphoid tissues showed moderate to severe follicular involution in addition to a variable degree of depletion of lymphocytes within the T-cell- dependent zones. Follicles were observed to be smaller in size and often showed a lack of visible germinal centers. No T-cell loss was observed during lymphocytic necrosis, except for several depleted splenic periarteriolar sheaths. Moderate numbers of large lymphoblasts were typically contained in T-cell zones. KFDV-infected macaques fractionally affected the GI tract (Kenyon et al., 1992).

There was a mucosal erosion leading to a reduced surface area of luminal epithelium in the stomach and large intestine in addition to villus blunting and ultimately fusion in the small intestine. Occasional crypt loss was observed in critically affected regions. A diffuse mild to moderate lymphohistiocytic infiltrate was observed to be present in the lamina propria and often seen to be accompanied by karyorrhectic debris with adjacent, intact, crypt epithelium. All KFD virus-infected macaques had moderate to marked, diffuse, fatty changes in the liver. Investigators also reported that KFD virus infections led to reduced numbers of peripheral blood lymphocytes in humans and primates. Thus, the data suggests *M. radiata* as a valuable model to study human disease caused by KFDV (Webb and Chatterjea, 1962).

### Rodent models

Rodents represent cheaper and more accessible models to study disease pathogeneses; however, they are less useful than bonnet macaques as suitable preclinical models for KFD. Following KFDV inoculation, mice have been reported to develop certain symptoms of KFD. The virus was first isolated from newborn (2–3 days old) mice after an intracranial inoculation of tissue or serum homogenates from dead NHPs (Work and Trapido, 1957). Further experiments revealed the development of severe disease in both young (3–4 weeks old) and adult (≥5 weeks of age) mice infected via intranasal, subcutaneous and intraperitoneal routes (Work, 1958; Nayar, 1972a,b). KFDV- infected mice (90–100%) succumbed to disease 7–9 days following infection, after a rapid disease progression marked by high viremia, clinical chemistry abnormalities and significant pathology in the brain and gastrointestinal tract (Dodd et al., 2014). Similar to published descriptions of human disease, the mice (especially the C57BL/6 mice) developed CBC abnormalities, hypoalbuminemia and elevated levels of blood urea nitrogen and liver transaminases (Pavri, 1989; Adhikari et al., 1993). The virus replicated in the brain and caused high levels of pro-inflammatory cytokine production but no viral load was detected in the visceral organs, except the lungs. Enlarged spleen and some evidence of hemorrhages in the liver were observed around day 9 post-infection. As compared to the human cases, the mice did not become febrile or showed overt symptoms of biphasic disease (Sawatsky et al., 2014). Also, despite developing chronic disease, the mice did not develop detectable neutralizing antibodies to the virus (Price, 1966). The presence of significant neuropathology and a rapid, lethal infection course, and the lack of marked spleen and liver pathology makes a mouse model a less predictive model of human KFD. Nonetheless, it has been employed to assess the protective effect of KFDV vaccines (Mansharamani and Dandawate, 1967).

## Diagnosis

Authentic and quick differential diagnostic test should be developed for the detection and confirmation of KFD as the clinical signs of KFD are indistinguishable from various other hemorrhagic/viral fevers. Hence, other diseases like influenza, typhoid, and rickettsial fevers should be differentially diagnosed from KFD (John et al., 2014). Various diagnostic tests were implemented initially for the detection of KFD including hemagglutination inhibition, neutralization tests, complement fixation and inoculation of serum obtained from patients into mice. Early diagnosis of KFD is better than late diagnosis as viremia elevates to 3 × 10^6^ CFU/ml within a few days of infection and remain that high for up to 2 weeks (Mourya et al., 2012). Due to the rapid progress in technologies, laboratories have developed different diagnostic methods (John et al., 2014).

### Molecular differential diagnostic laboratory testing

Different molecular tests such as real-time RT-PCR, IgG, and IgM capture ELISA [MAC-ELISA] have been developed in the BSL-3 lab for the detection and understanding of KFD (Mourya et al., 2012). Mourya et al. (2012) have reported the development of a nested RT-PCR [nRT-PCR] and a TaqMan-based real-time RT-PCR for early KFD diagnosis throughout the acute stage of infection. For the designing of primer, the specific NS-5 non-coding region of flaviviruses was made a target. Palacios et al. (2006) established Mass Tag polymerase chain reaction for the differential diagnosis of viruses causing VHFs. A version 1.0 VHF Greene MassTag Panel was established in which one of the targets was KFDV. The mass tags were identified by using mass spectrometry. Moreover, in a study performed in 2014, Multiplex One-Step Real-Time TaqMan qRT-PCR Assays were developed for the detection and quantification of about 28 viral pathogens (including KFDV) causing viral hemorrhagic fevers (Pang et al., 2014).

## Treatment and clinical management:

Timely hospitalization and supportive therapy are more important because no specific and well established antiviral treatment is available for KFD virus in humans currently. Maintenance of blood pressure, hydration and normal blood cell counts are the options included in supportive treatment (John et al., 2014). For secondary infections antipyretics, pain reliefs, antimicrobial therapy and blood transfusion are carried out while for nervous disorders, anticonvulsants, and corticosteroids are available (Adhikari et al., 1993). No approved antivirals exist for KFDV infection except for the vaccination and the prevention of tick bites.

On the basis of these findings, the frequently-used- IFN-α2a was evaluated but it was found to be lacking in limiting the KFDV virus titres. Other IFN- α/β was used at different concentrations but even though there were signs of reduced cellular damage, it was found that the replication of KFD was not sensitive to all the subtypes. Hence, the infectious titre is more reliable for the analyses of IFN as compared to the cytopathic effect (CPE) examining or monolayer staining. The KFDV has the capability to overcome the antiviral properties of IFN which was ascribed to the protein NS5. Hence, there is a need to evaluate other medication possibilities for people infected with KFD (Cook et al., 2016).

## Prevention and control of KFD

Due to the changes taking place in the resistance of acaricide, health policies for the public, environmental changes and development of new pathogen variants, a number of tick-borne diseases are emerging. To reverse them, proper measures are needed to be implemented. Prevention policies including timely diagnosis, quarantine, control of tick, and vaccination will act as a constraint to the spread of the virus to other regions. The virus that causes KFD is categorized as a risk group IV pathogen, and vaccination has been considered as one of the foremost strategies for the control of KFD (John et al., 2014). Formalin-inactivated, mouse-brain formulation of Russian spring-summer encephalitis virus (RSSEV) was the first vaccine made by Indian Council of Medical Research because of the nearly antigenic similarity between KFDV and RSSEV. However, it was discovered through studies that the immune response stimulated by the RSSEV vaccine is not that strong to combat and prevent the KFDV (Shah et al., 1962). Afterwards, in 1965, KFDV was prepared by first growing the KFDV in newborn Swiss albino mice brains and then inactivation with formalin (Mansharamani et al., 1965). Similarly, another vaccine for KFDV was developed by using the chick embryo for the growth of the virus but the vaccine was not strongly immunogenic and was unsuccessful in inducing an immune response in mice (Dandawate et al., 1965). Later on, in 1966, by using fibroblasts cultures of chick embryo as a growing medium for KFDV, a formalized vaccine was developed which was immunogenic, effective, safe and stable (Mansharamani and Dandawate, 1967; Mansharamani et al., 1967). Likewise, exertions were made to prepare a live attenuated vaccine. This was done by weakening the P9605 strain through the serial tissue culture passages (Bhatt and Anderson, 1971).

Kasabi et al. (2013a) conducted a study in affected regions in which the coverage of the vaccine was noticed to be very low and the effectiveness was about 62% in persons who were given first two doses and was around 83% in persons who were further given boosters. The prevalence of KFDV has also been found in the individuals who were even vaccinated against KFDV. Low coverage in affected areas, lack of proper conditions for storage and ignorance in boosters taking are the major shortcomings of the vaccines. Two-dose vaccine at an interlude of a month followed by boosters is the ongoing vaccination strategy in India. The first series is followed by a booster at 6–9 months and then successive boosters after every 5 years (Kasabi et al., 2013a).

Evidently, KFDV vaccines are being used on an extensive level, covering all the working risks units in the endemic areas, but still, for humans, it will remain a major problem. The population of birds and animals should be checked for the existence of KFD ticks (vectors) and the patterns of their migration should also be monitored by the radio tracking of wildlife (Ajesh et al., 2017). A study conducted by Bisson et al. (2015), highlighted the significance of timely detection of zoonotic diseases by monitoring animal mortality and morbidity. Through their research, they evaluated that out of 52% cases of dead and sick animals that were infected with the emerging diseases, just 9% were identified with animal mortality and morbidity prior to their spread in humans. Therefore, it was proposed that timely animal mortality and morbidity monitoring programme of both domesticated and wild animals infected with KFDV will aid in earlier detection and prevent the disease from spreading (Bisson et al., 2015).

In a study conducted by Patil et al. (2017), out of 76 cases, 13 were confirmed for KFD in the migrant workers, during harvesting of cashew nuts from the KFD infected areas in India (Patil et al., 2017). Infected ticks should be eradicated so as to control the spread of ticks in the whole forestry. A likely transmission of infection from a dead monkey to human can be prevented by use of insecticides in about a radius of 50 m circling the dead monkey. However, control programmes are difficult in practice as technically it is not feasible to carry a huge amount of water required for the spray of insecticide. Alternatively, tick-bite can be avoided by using repellents (Mourya et al., 2014).

In order to combat ticks, reduction of the source is also a chief control and preventive measure. Benzene hexachloride (BHC) has been discovered to be one of the most effective chemicals for about 6 weeks in the form of wettable triturate. All the local people of villages, wildlife professional photographers, travelers, camp personnel at forest must be counseled to use tick repellents such as NN-Diethyl-m-Tolumaide and Dimethyl phthalate (Roy et al., 2017). Moreover, to prevent direct contact with ticks, people should be instructed to use clothes having long sleeves (Kasabi et al., 2013b).

## Future perspectives

As KFDV is considered a risk group IV agent, there are considerable concerns regarding biosafety. Currently, KFDV from India and variant isolated from Saudi Arabia exist in restricted regions around the world (Mehla et al., 2009). This widespread distribution of KFDV across widely parted regions indicates the extensive mobility of KFDV (Roy et al., 2017). As a result of movement of rodents and monkeys which maintain the virus, KFDV possibly has penetrated to other newer regions. People who inhabit in forests and vicinities, in addition, those who work in the sanctuaries, parks, and reserve areas of forests stay at a higher risk of developing the infection. When enzootic infections occur and the sentinel wild animals (e.g., monkeys) begin to die, the incidence of the malady becomes evident. Establishing an incident based surveillance method for the monkey deaths in the sanctuaries of wildlife, national parks, the Western Ghats reserve forests and the nearby villages would assist in the early detection of the disease and thus would aid the institute in taking proper measures of control (Murhekar et al., 2015).

As the KFDV reservoir mainly exists in the jungle, controlling the disease emergence becomes an onerous task. Thus, it requires an approach for KFD that is interdisciplinary and a proper understanding of the vectors and their epidemiology is needed (Ajesh et al., 2017).

Currently, there is a dearth of co-infection studies linked to KFDV but the incidence of co-infection has recently been reported to be high among ticks (Moutailler et al., 2016) and there exists the possibility of KFDV being co-infecting ticks in synergism with other infectious agents. Further studies are expected to unravel the mystery of co-infection in ticks and the effect of co-infection on KFDV transmission.

Moreover, there is a need for control strategy establishment and firm administrative actions against deforestation and escalated the level of illicit demolishing of trees (Roy et al., 2017). For disease mapping, organizing sero-surveys in various districts of the areas seems remarkably promising (Hollidge et al., 2011). Similarly, in order to understand the evolution mode of virulence in KFDV, further molecular findings are required (Roy et al., 2017). More organized and large sample studies are needed to be focused on the molecular biology of KFDV, which could possibly lead to develop prophylactics and diagnostics in a prevailing way for the rapid eradication of the disease (Venkatesan et al., 2010).

For significantly reducing the spread of KFD in Karnataka five districts, which are endemic, formalin-inactivated tissue culture vaccine has been exploited since 1990. However, the immunity gained following vaccination is short lived; yearly boosters are suggested for about 5 successive years starting from the time when the latest case in the region was reported. Though the present vaccine in use is not very effectual, the effectiveness of the current vaccine can be determined by examining the genetic make-up of the present vaccine, derived from the initially isolated viral strain, as genetic diversity might have generated new strains of KFDV genetically divergent from the strain. Moreover, the degree of immunity that a particular vaccine gives could be indicated by thorough studies on the genetic makeup of the strains of KFDV from diverse geographical regions (Kasabi et al., 2013a).

For controlling KFD, future main concerns consist of considering the causes for low coverage of vaccine, assessing the effectiveness of vaccination policies, and evaluating long-lasting immunity provided by doses of annual boosters (Hollidge et al., 2011). Such type of understanding and consideration will lead toward the establishment of newer effective vaccines for KFD and prevention of the infection (Roy et al., 2017). Presently, for humans, no particular and well organized antiviral treatment is available; therefore further options for treatment needs to be assessed for individuals affected by KFD (Cook et al., 2016).

Efforts to produce anti-ticks vaccines have emanated for preventing multiple tick-borne infections. Identification and characterization of tick proteins that play a role in feeding and transmission of pathogen enables selection of suitable antigens to be used for anti-ticks vaccines (Sprong et al., 2014; Rodríguez-Mallon, 2016). Such vaccines, in addition to limiting tick feeding and reproduction, also hamper infection and pathogen transmission from tick to host (Merino et al., 2013). Further efforts not only to produce such vaccines but also to overcome limitations and evaluate the efficiency of these vaccines are required.

## Conclusion

Further in-depth analysis of KFDV and its pathogenesis is vital as it will pave the way for the development of better preventive and therapeutic approaches to counter KFD. Considering the contemporary status of vaccine availability for KFDV- low efficacy and use of vaccine not sanctioned by FDA- synthetic peptide vaccine can be considered for further development. The present multidimensional review covers various aspects of KFDV including epidemiology, molecular determinants of pathogenesis, ecological drivers, transmission, clinical manifestations, diagnosis, and control measures. Pathogenesis of this disease is not well understood mostly due to the lack of suitable animal models; henceforth, based on some published findings, a proposed pathogenesis model of KFD has been presented in this review. Finally, future perspectives are given to indicate further developments expected on KFD.

## Author contributions

SZS conceived the idea. The manuscript was written by SZS, BJ, AR, HN, and SN. The manuscript was edited by BJ. SZS, BJ, and IJ prepared and edited figures. ZR, SA, and NA contributed to language editing. All authors jointly proof-read manuscript. IJ, BJ, SZS, NA, SA and ZR rechecked and approved edited manuscript for publication.

### Conflict of interest statement

The authors declare that the research was conducted in the absence of any commercial or financial relationships that could be construed as a potential conflict of interest. The reviewer KL and handling Editor declared their shared affiliation.
